# Laboratory blood parameters and machine learning for the prognosis of esophageal squamous cell carcinoma

**DOI:** 10.3389/fonc.2024.1367008

**Published:** 2024-04-03

**Authors:** Feng Lu, Linlan Yang, Zhenglian Luo, Qiao He, Lijuan Shangguan, Mingfei Cao, Lichun Wu

**Affiliations:** ^1^ Department of Experimental Medicine, The People’s Hospital of Jianyang City, Jianyang, Sichuan, China; ^2^ College of Medical Technology, Chengdu University of Traditional Chinese Medicine, Chengdu, China; ^3^ Department of Transfusion Medicine, West China Hospital, Sichuan University, Chengdu, China; ^4^ Department of Clinical Laboratory, Sichuan Clinical Research Center for Cancer, Sichuan Cancer Hospital & Institute, Sichuan Cancer Center, Affiliated Cancer Hospital of University of Electronic Science and Technology of China, Chengdu, China; ^5^ Outpatient Department, People’s Hospital of Jianyang, Jianyang, Sichuan, China; ^6^ Department of Clinical Laboratory, Chuankong Hospital of Jianyang, Jianyang, Sichuan, China

**Keywords:** esophageal squamous cell carcinoma, machine learning, random survival forest, prognosis, laboratory blood parameters

## Abstract

**Background:**

In contemporary study, the death of esophageal squamous cell carcinoma (ESCC) patients need precise and expedient prognostic methodologies.

**Objective:**

To develop and validate a prognostic model tailored to ESCC patients, leveraging the power of machine learning (ML) techniques and drawing insights from comprehensive datasets of laboratory-derived blood parameters.

**Methods:**

Three ML approaches, including Gradient Boosting Machine (GBM), Random Survival Forest (RSF), and the classical Cox method, were employed to develop models on a dataset of 2521 ESCC patients with 27 features. The models were evaluated by concordance index (C-index) and time receiver operating characteristics (Time ROC) curves. We used the optimal model to evaluate the correlation between features and prognosis and divide patients into low- and high-risk groups by risk stratification. Its performance was analyzed by Kaplan-Meier curve and the comparison with AJCC8 stage. We further evaluate the comprehensive effectiveness of the model in ESCC subgroup by risk score and KDE (kernel density estimation) plotting.

**Results:**

RSF’s C-index (0.746) and AUC (three-year AUC 0.761, five-year AUC 0.771) had slight advantage over GBM and the classical Cox method. Subsequently, 14 features such as N stage, T stage, surgical margin, tumor length, age, Dissected LN number, MCH, Na, FIB, DBIL, CL, treatment, vascular invasion, and tumor grade were selected to build the model. Based on these, we found significant difference for survival rate between low-(3-year OS 81.8%, 5-year OS 69.8%) and high-risk (3-year OS 25.1%, 5-year OS 11.5%) patients in training set, which was also verified in test set (all P < 0.0001). Compared with the AJCC8th stage system, it showed a greater discriminative ability which is also in good agreement with its staging ability.

**Conclusion:**

We developed an ESCC prognostic model with good performance by clinical features and laboratory blood parameters.

## 1 Introduction

Esophageal cancer (EC), ranking as the sixth leading cause of cancer-related deaths worldwide, is one of the most common and highly aggressive malignancies (1). The one of the most prevalent subtypes of EC is esophageal squamous cell carcinoma (ESCC), accounting for about 90% cases in China (2, 3). With a five-year survival rate of less than 20%, the prognosis for ESCC patients, however, remains poor in advanced diagnostic techniques and treatment modalities (3–5). Therefore, early detection and accurate prognosis are crucial for improving patient outcomes.

Previous research has found that age, sex, tumor size, lymph node (LN) metastasis, tumor location, tumor invasion depth level, angiolymphatic invasion, pT stage and pN stage were known independent predictors of ESCC outcomes (6–9). Moreover, numerous of laboratory blood parameters, including mean platelet volume(MPV), hemoglobin (HB), C-reactive protein to albumin ratio (CAR), neutrophil-lymphocyte ratio (NLR), platelet-lymphocyte ratio (PLR), monocyte-lymphocyte ratio (MLR), systemic immune-inflammation index (SII) and prognostic nutritional index (PNI), have been shown to be related to tumor progression and unfavorable prognosis of ESCC (10–13). Despite the availability of these factors, it remains uncertain whether they should be collectively considered to establish and apply a survival prediction model for ESCC. Machine learning (ML), a field within artificial intelligence, has emerged as a promising tool for enhancing the prognosis and management of various cancers, offering the potential to revolutionize clinical decision-making (14–16). ML algorithms can analyze large, complex datasets to identify patterns and relationships that may not be readily apparent to human observers (17). By leveraging these computational techniques, researchers have the potential to develop more accurate and personalized prognostic models for ESCC patients. For example, researchers have used ML algorithms to identify protein markers of tissue microarrays and immunohistochemistry, and constructed a new ESCC staging system MASAN to predict the survival of ESCC patients. They found that the ML model showed better prognostic prediction accuracy compared with the currently used TNM staging system (18). Additionally, ML has been employed to analyze imaging data, such as computed tomography (CT) scans. They developed a ML model that combined radiomics and clinical features to predict the survival of ESCC patients and found the ML model performed better than radiomics or clinical alone and achieved high accuracy, which was able to accurate predict 3 years progression free survival (PFS) and overall survival (OS) of non-surgical ESCC patients (19).

Despite ML technology is widely used in the cancer research, especially in the survival prediction of ESCC, most of the research was focused on genetic data (16, 20, 21). The research on the construction of machine learning models bases on real-world laboratory data is extremely rare to find that can be used to predict the prognosis of ESCC. Developing a robust predictive model for ESCC could assist in selecting high-risk patients for personalized and intensified treatment based on risk stratification, as well as identifying candidates for active follow-up. In this paper, we aim to analyze the prognostic significance of various clinicopathologic factors and laboratory index in patients with ESCC patients and attempted to construct a more accurate and personalized prognostic model for ESCC patients, ultimately improving their clinical outcomes and quality of life.

## 2 Materials and methods

### 2.1 Patients

A total of 2521 consecutive patients who underwent esophagus surgery with or without postoperative radiotherapy at Sichuan Cancer Hospital between January 2009 and December 2017 were included in this study. Medical data of ESCC patients were collected from the electronic medical record system of Sichuan cancer hospital, and they were followed up to obtain prognosis and survival status. The inclusion criteria were: (1) post-histological confirmation of ESCC without distant metastasis, (2) non-cervical esophageal cancer, (3) no previous anticancer therapy, and (4) complete clinical, blood parameter, and follow-up data. The exclusion criteria were: (1) history of other malignancies or perioperative mortality, (2) invasion of the neck with cancer, (3) incomplete follow-up information, and (4) follow-up period less than 3 months.

Patients who enrolled in the study were randomly split into the training set and test set by 7:3. The training set was employed to model development and tune parameters, and the testing set was applied to model validation. All patients included in the study were staged using the American Joint Committee on Cancer (AJCC) 8th edition TNM classification system.

### 2.2 Features and outcomes

Among eligible cases, we selected 27 predictors, which include patient clinicopathological characteristics, laboratory indicators, and survival outcomes from medical records. These predictors are as follows: clinicopathological characteristics such as age, sex, Karnofsky performance scale (KPS) score, tumor length, tumor grade, tumor location, vascular invasion, surgical margin, dissected lymph nodes (LN) number, nerve invasion, T stage, N stage, AJCC8th stage, and treatment; laboratory indicators such as Mean Corpuscular Hemoglobin (MCH), Mean Platelet Volume (MPV), Direct Bilirubin (DBIL), Albumin (ALB), Gamma-glutamyl Transferase (GGT), Sodium (Na), Chlorine (Cl), Magnesium (Mg), Fibrinogen (FIB), Neutrophil-to-lymphocyte ratio (NLR), Monocytes-to-lymphocyte ratio (MLR), platelet‐to‐lymphocyte ratio (PLR), and Systemic immune‐inflammation index (SII). The model’s predictive capability was gauged in terms of 1, 3, and 5-year overall survival (OS), which was calculated as the time from the date of surgery until either death or the last follow-up.

### 2.3 Model development and validation

Three survival analysis methods, including GBM, RSF, and the classical Cox method, were used for constructing survival prediction models. Hyper-parameter of each model was tuned by using 10-fold cross-validation. The concordance index(C-index) and the time receiver operating characteristic (timeROC) curve were employed to evaluate the performance of survival prediction model, and the model with the best performance was selected for further research.

To enhance the clinical application of the model, we utilized the prognostic model to calculate the risk score for disease progression. Based on the optimal cutoff value for the risk score, patients were stratified into high- and low-risk groups. Additionally, we employed Kaplan-Meier curves using the R “survminer” package to assess survival probabilities in different risk stratification patient groups.

### 2.4 Statistical analysis

Patient clinical parameters, pathologic information and laboratory data were analyzed by using R software 4.2.3 (https://www.r-project.org/). The continuous data are expressed as the median (interquartile range [IQR]) or the mean ± standard deviation (SD), while the categorical data are presented as numbers (percentages). For two-group comparisons, the unpaired t-test or the Mann-Whitney U test or the Chi-square test were used, depending on the circumstances. The Kaplan–Meier curves and the log-rank test were utilized to compare the cumulative survival rates of different patient subgroups. The adjusted risk estimates (hazard ratio or HR and its 95% confidence interval or 95% CI) for mortality were calculated using the multivariate Weibull proportional hazards regression analysis. Values of p < 0.05 were regarded as statistically significant.

### 2.5 Ethical approval and consent to participate

This study (Grant No. SCCHEC-02-2020-015) received approval from the ethics committee of Sichuan Cancer Hospital and was conducted in accordance with the Guidelines for Good Clinical Practice and the Declaration of Helsinki. As the study was retrospective in nature, the ethics committee of Sichuan Cancer Hospital waived the requirement for informed consent.

## 3 Results

### 3.1 Clinicopathological characteristics

**Figure 1:**
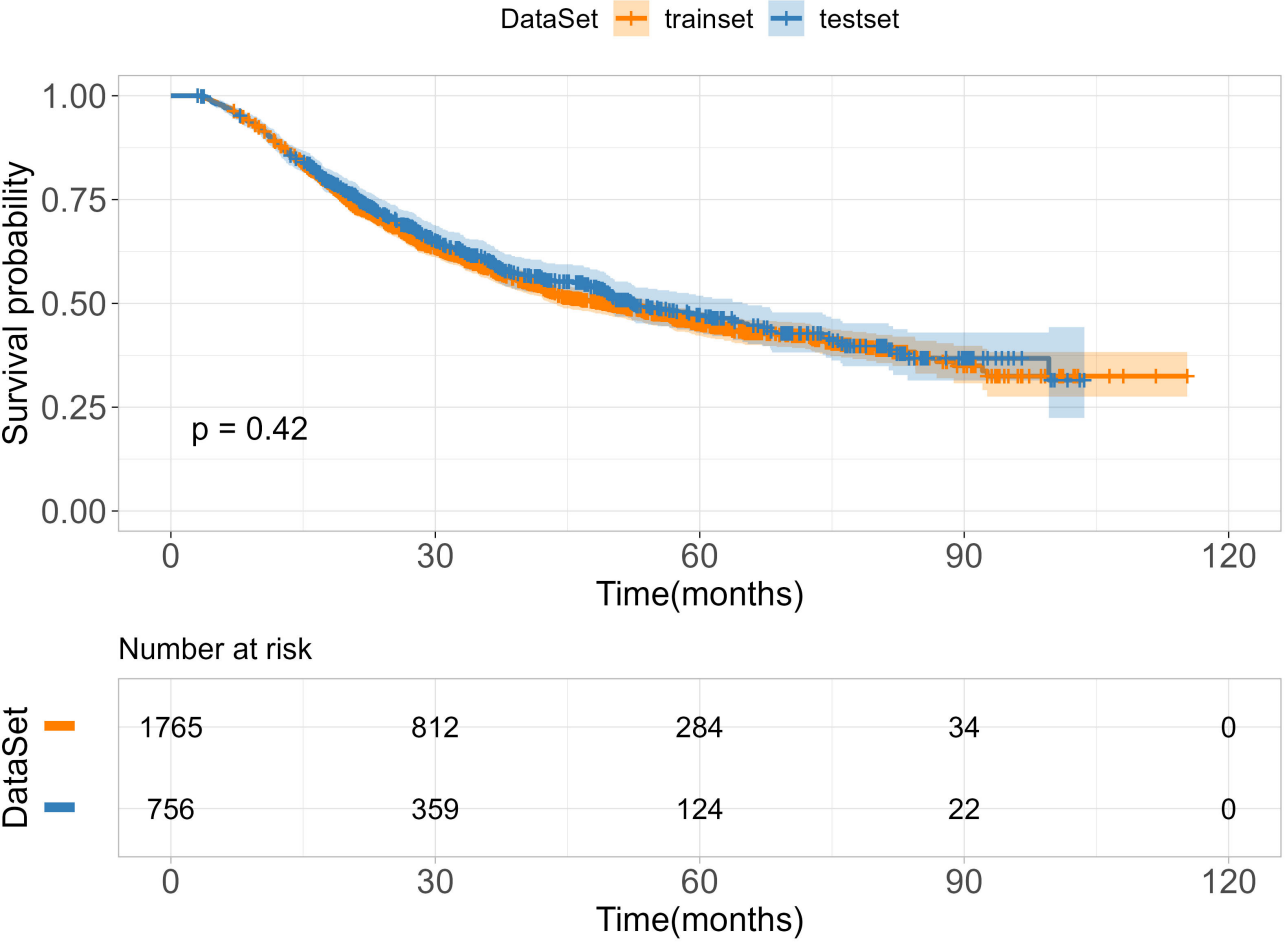
Survival curves of the training and test set.

A total of 2521 patients with ESCC were included in this study. Of these, 1765 patients were allocated to the training cohort, while 756 patients were assigned to the test cohort. There was no significant difference between the training and test sets (p > 0.05), as summarized in **Table 1**. The median age of included patients was 62.0-years old (range, 57-67years) in the training cohort and 62.0-years old (range, 56-67years) in the validation cohort. The median survival times of OS were 27.5 months (range,17.6–48.8 months) in training cohort and 28.5 months (range,18.0–49.4 months) in test cohort. The time range of follow-up is 3.0-115.3 months. In addition, we employed the Kaplan-Meier method to assess the disparity in survival probabilities between the training and test datasets. Our findings indicate that there is no significant difference in overall survival rates between the two datasets (**Figure 1**, P=0.42).

**Table 1:** Summary descriptives table by groups of `DataSet’.

	[ALL]	trainset	testset	p.overall
	*N=2521*	*N=1765*	*N=756*	
age	62.0 [57.0;67.0]	62.0 [57.0;67.0]	62.0 [56.0;67.0]	0.092
treatment:				0.235
Surgery	1337 (53.0%)	948 (53.7%)	389 (51.5%)	
Surgery+RT	50 (1.98%)	40 (2.27%)	10 (1.32%)	
Surgery+CT	852 (33.8%)	581 (32.9%)	271 (35.8%)	
Surgery+CCRT	282 (11.2%)	196 (11.1%)	86 (11.4%)	
Sex:				0.907
male	2059 (81.7%)	1440 (81.6%)	619 (81.9%)	
female	462 (18.3%)	325 (18.4%)	137 (18.1%)	
KPS_score:				0.852
90-100	1436 (57.0%)	1008 (57.1%)	428 (56.6%)	
70-80	1085 (43.0%)	757 (42.9%)	328 (43.4%)	
Tumor_length	4.00 [2.80;5.00]	4.00 [3.00;5.00]	3.65 [2.50;5.00]	0.161
Tumor_Grade:				0.409
Well_differentiated	537 (21.3%)	375 (21.2%)	162 (21.4%)	
Moderate_differentiation	1000 (39.7%)	687 (38.9%)	313 (41.4%)	
Poorly_differentiated	984 (39.0%)	703 (39.8%)	281 (37.2%)	
tumor_location:				0.405
Lower_chest	544 (21.6%)	369 (20.9%)	175 (23.1%)	
Middle_chest	1366 (54.2%)	960 (54.4%)	406 (53.7%)	
Upper_chest	611 (24.2%)	436 (24.7%)	175 (23.1%)	
Surgical_margin:				0.674
R0	2402 (95.3%)	1686 (95.5%)	716 (94.7%)	
R1	78 (3.09%)	52 (2.95%)	26 (3.44%)	
R2	41 (1.63%)	27 (1.53%)	14 (1.85%)	
Vascular_invasion:				0.159
no	2085 (82.7%)	1447 (82.0%)	638 (84.4%)	
yes	436 (17.3%)	318 (18.0%)	118 (15.6%)	
Nerve_invasion:				0.899
no	2042 (81.0%)	1428 (80.9%)	614 (81.2%)	
yes	479 (19.0%)	337 (19.1%)	142 (18.8%)	
Dissected_LN_number	20.0 [14.0;28.0]	20.0 [13.0;28.0]	20.0 [14.0;28.0]	0.387
T_stage:				0.063
T1	311 (12.3%)	214 (12.1%)	97 (12.8%)	
T2	491 (19.5%)	364 (20.6%)	127 (16.8%)	
T3	1490 (59.1%)	1019 (57.7%)	471 (62.3%)	
T4	229 (9.08%)	168 (9.52%)	61 (8.07%)	
N_stage:				0.145
N0	1138 (45.1%)	791 (44.8%)	347 (45.9%)	
N1	734 (29.1%)	512 (29.0%)	222 (29.4%)	
N2	430 (17.1%)	294 (16.7%)	136 (18.0%)	
N3	219 (8.69%)	168 (9.52%)	51 (6.75%)	
AJCC8th_stage:				0.474
I	303 (12.0%)	216 (12.2%)	87 (11.5%)	
II	816 (32.4%)	563 (31.9%)	253 (33.5%)	
III	1104 (43.8%)	767 (43.5%)	337 (44.6%)	
IV	298 (11.8%)	219 (12.4%)	79 (10.4%)	
MCH	31.4 [30.0;32.7]	31.4 [30.1;32.6]	31.5 [30.0;32.8]	0.840
MPV	11.5 [10.3;12.7]	11.5 [10.3;12.7]	11.6 [10.3;12.7]	0.957
DBIL	4.87 [3.60;6.39]	4.78 [3.60;6.34]	5.00 [3.79;6.50]	0.060
ALB	42.9 [40.4;45.2]	42.8 [40.3;45.1]	43.1 [40.6;45.3]	0.090
GGT	4.58 [4.10;5.19]	4.59 [4.09;5.21]	4.55 [4.10;5.16]	0.604
Na	141 [139;142]	141 [139;142]	141 [139;143]	0.362
CL	104 [102;106]	104 [103;106]	104 [102;106]	0.123
Mg	0.95 [0.88;1.01]	0.95 [0.88;1.01]	0.95 [0.89;1.01]	0.699
FIB	3.21 [2.67;3.82]	3.20 [2.67;3.82]	3.22 [2.67;3.83]	0.700
NLR	2.54 [1.86;3.50]	2.55 [1.87;3.50]	2.50 [1.82;3.56]	0.823

### 3.2 Machine learning model development

**Figure 2:**
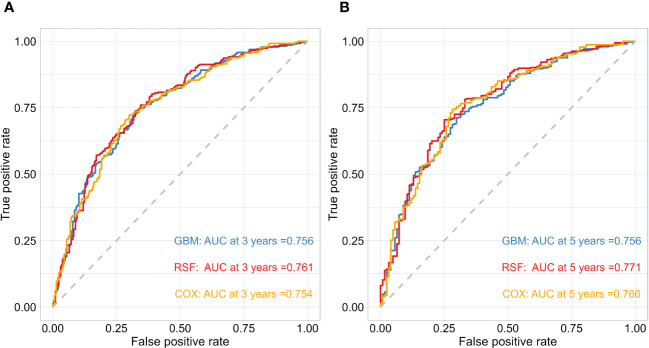
The performance of the risk model was compared using time-dependent ROC curves at 3 **(A)** and 5-year **(B)** follow-up times in test cohorts.

After obtaining the optimal parameters for each model, we trained them using the training set and evaluated their performance on the test set using the C-index and Time-dependent AUC metrics. **Table 2** displays the C-index results for each model, with GBM, RSF, and Cox achieving scores of 0.744, 0.746, and 0.741, respectively. Additionally, **Figure 2** depicts the time-dependent ROC curves for each model at 3-year and 5-year intervals. The 3-year AUC values for GBM, RSF, and Cox were 0.756, 0.761, and 0.754, respectively, while the 5-year AUC values were 0.756, 0.771, and 0.766, respectively. Based on these results, the RSF model exhibits a slight advantage over GBM and Cox in terms of both the C-index and time-dependent AUC. Consequently, we will use the RSF method to identify the best survival prediction model for further experimentation, including feature importance and model applications.

**Table 2:** The performance of the machine learn methods.

Methods	C-index	SD
GBM	0.744	0.031
RSF	0.746	0.031
Cox	0.741	0.030

### 3.3 Evaluation of feature importance

**Figure 3:**
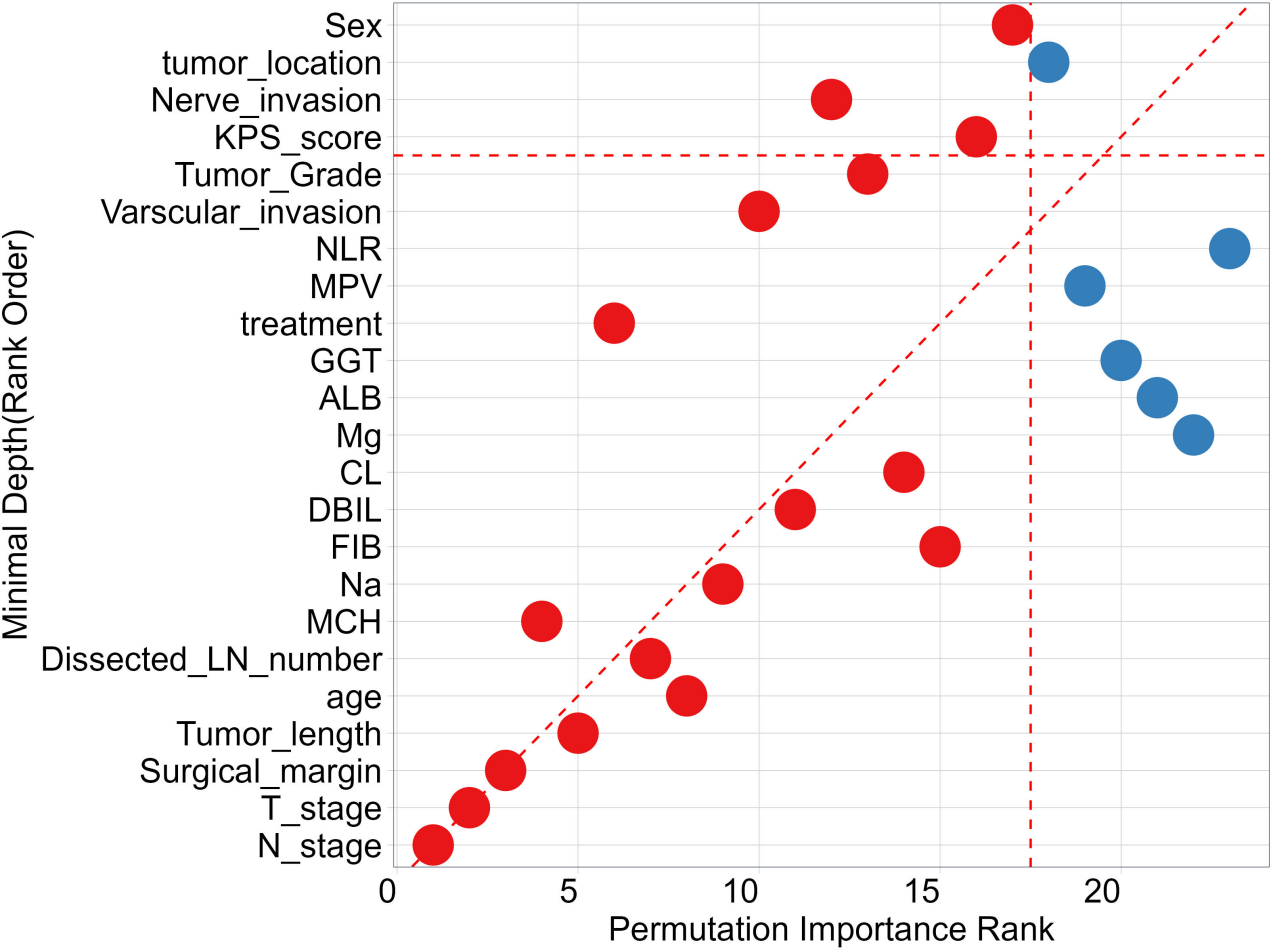
Comparing Minimal Depth and permutation importance ranks. Red points indicate positive permutation importance, blue indicates negative permutation importance. Points on the red dashed line are ranked equivalently, points above have higher permutation importance ranking, those below have higher minimal depth ranking.

**Figure 4:**
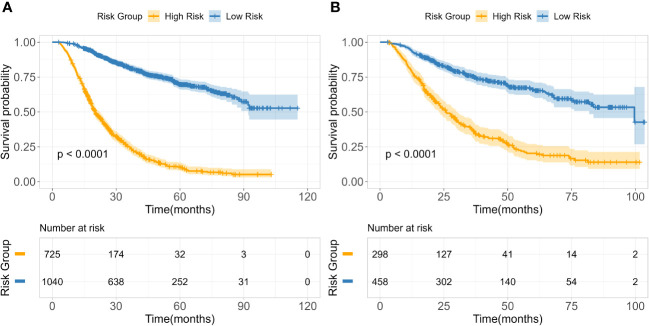
Kaplan-Meier survival curves in the training **(A)** and test **(B)** cohorts.

To identify features that significantly impact the disease progression of ESCC patients, we assess the significance of 27 features using minimal depth and permutation importance methods within the RSF model. The minimal depth metric is utilized to assess the impact of significant risk factors by determining the average initial split depth for each variable across the entire forest of trees. The underlying assumption of this metric is that lower minimal depth values denote variables that effectively segregate sizable clusters of observations, thereby exerting a substantial influence on the forest prediction. The permutation importance is determined by comparing the out-of-bag (OOB) prediction error before and after randomly permuting the values of each variable. A high value of variable importance indicates that the variable plays a crucial role in the predictive accuracy of the forest, while a variable importance score close to zero indicates that the variable has minimal impact on predictive accuracy. Since the permutation importance and minimal depth measures utilize distinct criteria, we expect the ranking of variables to vary slightly. We compared the rankings between minimal depth and permutation importance in **Figure 3**. We can observe that the two methods have almost identical results in selecting the most important variable, which are N stage, T stage and surgical margin. Although the rankings of other variables differ, the differences are minor. By combining the variable selection results of the two methods, 14 important features including N stage, T stage, surgical margin, tumor length, age, Dissected LN number, MCH, Na, FIB, DBIL, CL, treatment, vascular invasion, and tumor grade are important variables that meet the criteria of both methods and have a significant impact on the survival outcome of ESCC patients. Regardless of the method used, tumor location is not an important variable that determines the survival outcome of ESCC patients. In addition, we also discuss the relationship between each single feature and model survival prediction: N stage and T stage and surgical margin feature still have the best survival prediction ability, while laboratory parameters show similar prediction ability in **Figure 4**.

### 3.4 Application of survival prediction model

Based on 14 feature variables, we constructed the RSF model. To investigate the predictive accuracy of the RSF model for disease progression, we utilized the optimal cutoff value to classify patients into high- and low-risk groups. The Kaplan-Meier survival curves for these two risk groups are presented in **Figure 4**. Notably, there were significant differences in survival rates between the high- and low-risk subgroups in both the training and test cohorts (all p<0.0001). The risk stratification system predicted the 3-year overall survival probabilities for low-, and high-risk subgroups were 81.8%, and 25.1% in the training cohort, and were 75.4%, and 35.6% in the test cohort, respectively. Furthermore, the risk stratification predicted 5-year overall survival probabilities for the low- and high-risk subgroups were 69.8% and 11.5% in the training cohort, and 66.0% and 20.3% in the test cohort, respectively (**Table 3**). These findings demonstrate that the RSF prognostic model exhibits strong discriminatory ability in predicting outcomes for ESCC patients.

**Table 3:** 3,5-year OS survival probability of RSF model-based risk stratification in training and validation cohorts.

Risk groups	Training cohort		Test cohort	
	3-year survival	5-year survival	3-year survival	5-year survival
Low risk	81.8%(79.2~84.4)	69.8%(66.3~73.5)	75.4%(71.3~79.9)	66.0%(60.8~71.6)
High risk	25.1%(21.8~28.8)	11.5%(8.05~13.8)	35.6%(32.0~44.0)	20.3%(15.3~27.0)

**Figure 5:**
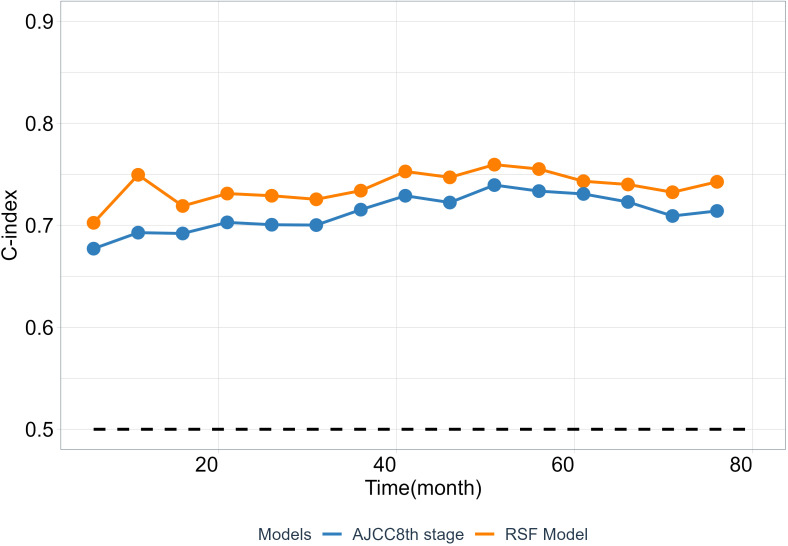
The predictability of RSF model and AJCC8th stage. The C-index at different time points was calculated from the validation cohort.

As is well known that the 8th TNM staging system (AJCC8th stage) is an important and widely used prognostic factor, has a huge impact on ESCC patients’ future survival state. To test the clinical practice and generalization ability of the model, we employed the C-index to compare the performance between the RSF model and the AJCC8th stage to examined whether the RSF model provide better predictive value than the AJCC8th stage system. The results show that RSF model has a greater discriminative ability than the AJCC8th stage system at different time points from the validation set (**Figure 5**).

### 3.5 Subgroup analysis of survival prediction model

**Figure 6:**
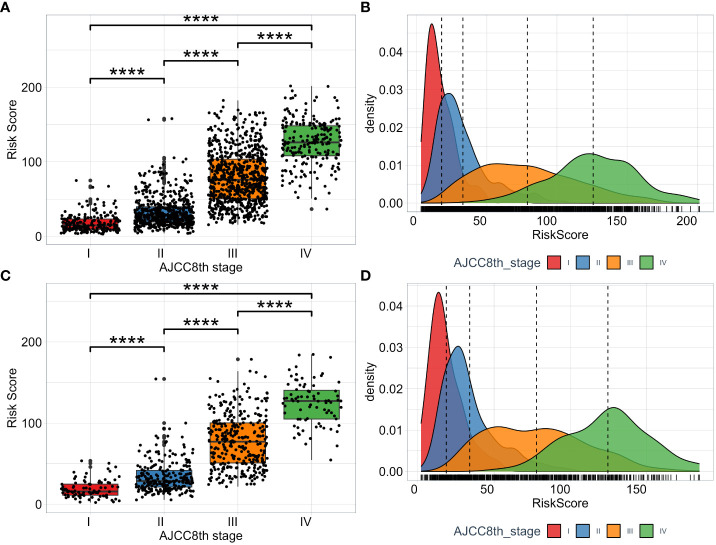
The distribution of RSF risk scores among subgroups of TNM AJCC8th stage. The risk scores in the training set **(A)** and test set **(C)**. The KDE density plotting of risk score distribution in the training set **(B)** and test set **(D)**.

To fully evaluate the model’s performance, we investigate and compare the distribution of risk scores calculated by the model across different AJCC8th TNM stage subgroups. The risk score among the subgroups of AJCC8th TNM stage is significant difference (**Figures 6A, C**). From stage I to stage IV, we can see that as the tumor stage increases, the estimated risk score from this model also increases, which is consistent with clinical experience. Moreover, we visualize the distribution of risk scores of each TNM stage subgroup by utilizing the KDE (Kernel Density Estimation) plotting. The results shown in (**Figures 6B, D**). The model has the ability to capture and assess the differences in the risk of disease progression among different TNM stage subgroups. While the TNM stage is a crucial factor in predicting prognosis, (**Figures 6B, D**) shows that there is overlap in the areas under the density curves, indicating that the interaction of multiple factors, including tumor stage, can result in complex prognostic predictions.

## 4 Discussion

As a kind of cancer with poor prognosis, accurate prognosis detection is very important for the survival rate of patients with esophageal cancer after treatment. Despite some studies have constructed prognostic models for ESCC, most of the experiments are still based on genetic studies, and there are few prognostic models based on real clinical data. Therefore, we constructed an ESCC prognostic model with patients’ clinicopathological factors and laboratory indicators.

In this experiment, we select the appropriate model through c-index and timeROC, and then select the features that can be included, and detect. The results show that the ESCC prognostic model we constructed has a better prognosis than the classical AJCC8 model. At the same time, this model has more advantages than the traditional TNM staging. The model divides patients into low-, high-risk, and low-risk groups with more patients and higher OS. This means that the model can provide better guidance for clinical treatment and avoid overtreatment to a great extent. Not only that, the risk scores of different TNM stages also show the differences of the model among different subgroups, which indicates that the ESCC classification may be more accurate under this model.

Compared with some previous studies, the prognostic ability of our model (three-year AUC 0.761, five-year AUC 0.771) also showed some advantages. In Feng’s experiment (10), they used laboratory data such as HB and CAR to construct a COHCP model to evaluate the prognosis of ESCC, and its AUC (0.771 for continuous and 0.744 for categorical) showed good predictive ability. In another study, Jayaprakasam’s team (22) constructed a prognostic model of ESCC using PET and CT, with an AUC of 0. 73. Zhang et al (23) also used LncRNA to predict the prognosis of ESCC, the AUC values of the model in one year, three years and five years were 0.670, 0.749 and 0.757, respectively.

**Figure 7:**
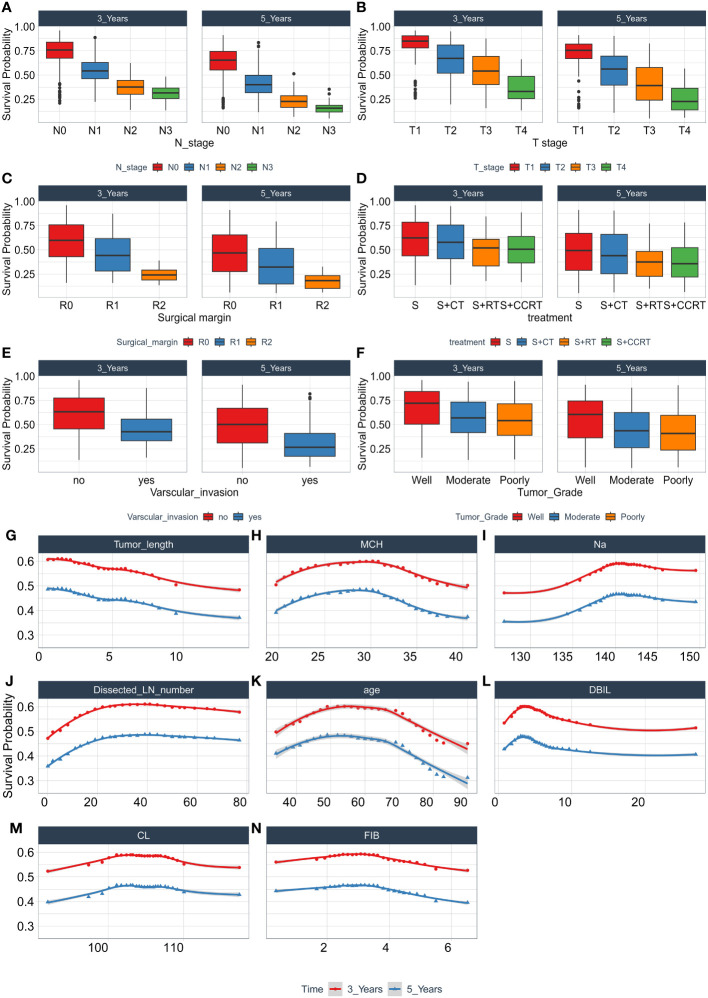
The relationship between each single feature and model survival prediction. Abscissa is the quantitative and semi-quantitative results of each feature, and ordinate is the survival rate. Including the survival analysis of N stage **(A)**, T stage **(B)**, Surgical margin **(C)**, Treatment **(D)**, Varscular invasion **(E)**, Tumor Grade **(F)**, Tumor length **(G)**, MCH **(H)**, Na **(I)**, Dissected LN number **(J)**, age **(K)**, DBIL **(L)**, CL **(M)**, FIB **(N)**, and evaluating its three-year and five-year survival rate.

During the study, we use the combination of clinical factors and experimental data, which greatly enhance the clinical convenience and save a lot of cost compared with conventional gene testing. Through permutation importance and Minimal Depth, 14 important features including N stage, T stage, surgical margin, tumor length, age, Dissected LN number, MCH, Na, FIB, DBIL, CL, treatment, vascular invasion, and tumor grade were screened out. It was found that these factors, as important predictors of OS in patients with ESCC, made a high contribution to the construction of predictive models, and the prognostic models based on the above 14 features were also more reliable. Among these 14 characteristics, N stage (**Figure 7A**) and T stage (**Figure 7B**) have been widely regarded as prognostic factors in patients with ESCC, and their importance as an important independent predictor of EC patients in this study cannot be ignored. Surgical treatment is the main treatment for patients with ESCC, the residual cancer cells at the incisal margin is the main cause of recurrence, and the prediction of ESCC is also very important (**Figure 7C**). Tumor length (**Figure 7G**), dissected LN number (**Figure 7J**), vascular invasion (**Figure 7E**) and tumor grade (**Figure 7F**) are closely related to the progression of cancer and are routine prognostic factors. Although age (**Figure 7K**) is controversial for the prognosis of patients with ESCC, it is a good predictor in this experiment. Studies have shown that the nutritional status of patients is also closely related to the prognosis of tumors (10), and MCH, Na, FIB, DBIL, CL (**Figures 7H, I, L–N**) and other factors also show high predictive ability in this experiment. In addition, the prognosis of patients with ESCC varies with different treatments, and treatment (**Figure 7D**) (24) is also included in this model.

However, it is worth noting that the samples in this experiment are from Sichuan Cancer Hospital, and the patients covered are from local areas of China. Whether the model can be used in other regions or ethnic groups still needs further research.

## Data availability statement

The original contributions presented in the study are included in the article/supplementary material, further inquiries can be directed to the corresponding author/s.

## Ethics statement

This study (Grant No. SCCHEC-02-2020-015) received approval from the ethics committee of Sichuan Cancer Hospital and was conducted in compliance with the Guidelines for Good Clinical Practice and the Declaration of Helsinki. The studies were conducted in accordance with the local legislation and institutional requirements. The ethics committee/institutional review board waived the requirement of written informed consent for participation from the participants or the participants’ legal guardians/next of kin because due to the retrospective nature of the study, the informed consent requirement was waived by the ethics committee of Sichuan Cancer Hospital. Written informed consent was not obtained from the individual(s) for the publication of any potentially identifiable images or data included in this article because Due to the retrospective nature of the study, the informed consent requirement was waived by the ethics committee of Sichuan Cancer Hospital.

## Author contributions

FL: Funding acquisition, Project administration, Writing – review & editing. LY: Conceptualization, Investigation, Writing – original draft. ZL: Data curation, Investigation, Writing – review & editing. QH: Methodology, Software, Supervision, Writing – review & editing. LS: Formal analysis, Writing – review & editing. MC: Resources, Visualization, Writing – review & editing. LW: Data curation, Visualization, Writing – original draft.
